# Case Report: A Case of Trimethoprim/Sulfamethoxazole-Triggered Hypotensive Shock: Cytokine Release Syndrome Related to Immune Checkpoint Inhibitors and Drug-Induced Hypersensitivity Syndrome

**DOI:** 10.3389/fonc.2021.681997

**Published:** 2021-04-30

**Authors:** Tetsuya Urasaki, Makiko Ono, Toshiaki Mochizuki, Koichi Takeda, Aya Nishizawa, Eri Fukagawa, Motohiro Fujiwara, Yoshinobu Komai, Shigehisa Kitano, Takeshi Yuasa, Junji Yonese, Shunji Takahashi

**Affiliations:** ^1^ Department of Medical Oncology, Cancer Institute Hospital of Japanese Foundation for Cancer Research, Tokyo, Japan; ^2^ Department of Emergency Medicine, Cancer Institute Hospital of Japanese Foundation for Cancer Research, Tokyo, Japan; ^3^ Department of Infectious Diseases, Cancer Institute Hospital of Japanese Foundation for Cancer Research, Tokyo, Japan; ^4^ Department of Dermatology, Cancer Institute Hospital of Japanese Foundation for Cancer Research, Tokyo, Japan; ^5^ Department of Urology, Cancer Institute Hospital of Japanese Foundation for Cancer Research, Tokyo, Japan; ^6^ Division of Cancer Immunotherapy Development, Advanced Medical Development Center, Cancer Institute Hospital of Japanese Foundation for Cancer Research, Tokyo, Japan

**Keywords:** immune checkpoint inhibitor, immune-related adverse event, trimethoprim/sulfamethoxazole, cytokine release syndrome, drug-induced hypersensitivity syndrome

## Abstract

Currently, only a few reports exist on the cytokine release syndrome (CRS) as one of the severe immune-related adverse events (irAEs) induced by immune checkpoint inhibitors (ICIs). Notably, it is very rare that grade 4 CRS related to ICI therapy overlaps with the drug-induced hypersensitivity syndrome (DiHS). A 46-year old woman with metastatic kidney cancer had grade 3 interstitial pneumonitis induced by four cycles of combination therapy of anti-programmed death-1 and anti-cytotoxic T lymphocyte-4 antibodies after right cytoreductive nephrectomy. Prophylactic administration of trimethoprim/sulfamethoxazole (TMP/SMX) was started concomitantly with prednisolone therapy to treat the interstitial pneumonitis. She developed hypotensive shock when reducing the dosage of prednisolone, and required intubation and ventilation using vasopressors at the intensive care unit. She subsequently exhibited prominent leukocytosis and an increased level of C-reactive protein, suggesting markedly increased cytokine levels. Interestingly, facial edema and erythema increased in association with pyrexia, leukocytosis, liver dysfunction, and renal failure, suggesting that she developed DiHS. She received hemodialysis three times, a plasma exchange, and anti-interleukin-6 therapy to treat severe renal dysfunction, a thrombotic thrombocytopenic purpura-suspected condition, and possible grade 4 CRS, respectively. Although these therapies did not elicit sufficient effects, high-dose administration of intravenous immunoglobulin was successful. With steroid mini-pulse therapy and the subsequent administration of prednisolone, she recovered successfully. To the best of our knowledge, this is the first report that ICIs and TMP/SMX can induce hypotensive shock accompanied with CRS and DiHS during immunosuppressive therapy for an irAE. Importantly, the prophylactic administration of TMP/SMX should be performed cautiously to avoid severe drug reactions such as CRS or DiHS.

## Introduction

Immune checkpoint inhibitors (ICIs) are efficacious for a variety of refractory or relapsed cancers; however, ICIs can unexpectedly induce various immune-related adverse events (irAEs) in different organs. Cytokine release syndrome (CRS) can occur as an irAE, although the severe type is considered to be very rare. Immunosuppression therapy for irAEs is usually performed using corticosteroids with prophylactic administration of trimethoprim/sulfamethoxazole (TMP/SMX) to prevent Pneumocystis pneumonia, a common and lethal infection in patients receiving high-dose corticosteroid therapy. Although some guidelines for managing ICI-related toxicities recommended that prophylactic antibiotics should be administered to prevent opportunistic infections in patients under long-term immunosuppressive medication, when oral TMP/SMX administration should be initiated has not been clarified. TMP/SMX, which causes fever and rashes frequently, is also known to rarely evoke the drug-induced hypersensitivity syndrome (DiHS) (1), which is a life-threatening multiorgan system reaction.

## Case Presentation

A 46-year-old woman with metastatic clear cell renal cell carcinoma had hypotensive shock with a 12-day history of high-dose prednisolone administration for interstitial pneumonitis induced by combination therapy of ICIs. She had no significant medical history. She had received 4 cycles of nivolumab, an anti-programmed cell death-1 monoclonal antibody (3 mg/kg) and ipilimumab, an anti-cytotoxic T lymphocyte antigen-4 monoclonal antibody (1 mg/kg) combination therapy for metastatic pulmonary tumors after right cytoreductive nephrectomy. Eleven days after the last administration of nivolumab and ipilimumab, she felt short of breath with a progressive dry cough. A computed tomography scan revealed ground-glass opacities in the peripheral fields of bilateral lungs and she had concomitant hypoxemia, resulting in a diagnosis of grade 3 interstitial pneumonitis. She was started on intravenous prednisolone (100 mg/day [2 mg/kg/day]) concurrently with prophylactic daily trimethoprim/sulfamethoxazole (TMP/SMX; 80/400 mg, respectively). These treatments improved the immune-related interstitial pneumonitis and dyspnea. Ten days after beginning corticosteroid therapy, her condition almost fully resolved and a computed tomography image of the bilateral lungs indicated a good response to the corticosteroid therapy. Accordingly, the dose of prednisolone was reduced from 100 to 70 mg/day (from 2 to 1.4 mg/kg/day).

**Figure 1:**
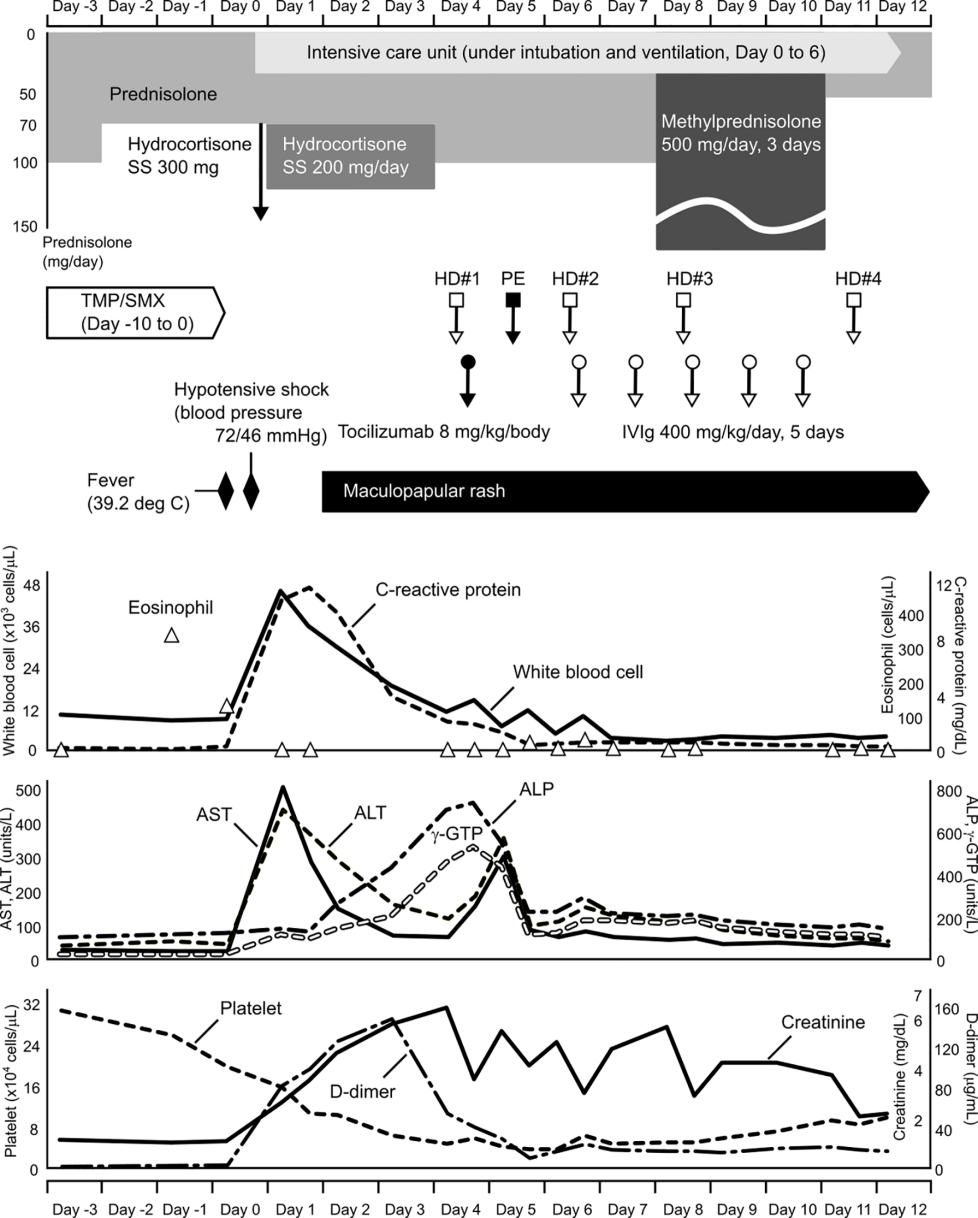
Clinical course before and after treatment for hypotensive shock. Time flows from left to right, and corresponding information on changes in clinical laboratory data are aligned vertically. SS, sodium succinate; TMP/SMX, trimethoprim/sulfamethoxazole; HD, hemodialysis; PE, plasma exchange; IVIg, intravenous immunoglobulin; AST, aspartate aminotransferase; ALT, alanine aminotransaminase; ALP, alkaline phosphatase; γ-GTP, gamma-glutamyl transpeptidase.

**Figure 2:**
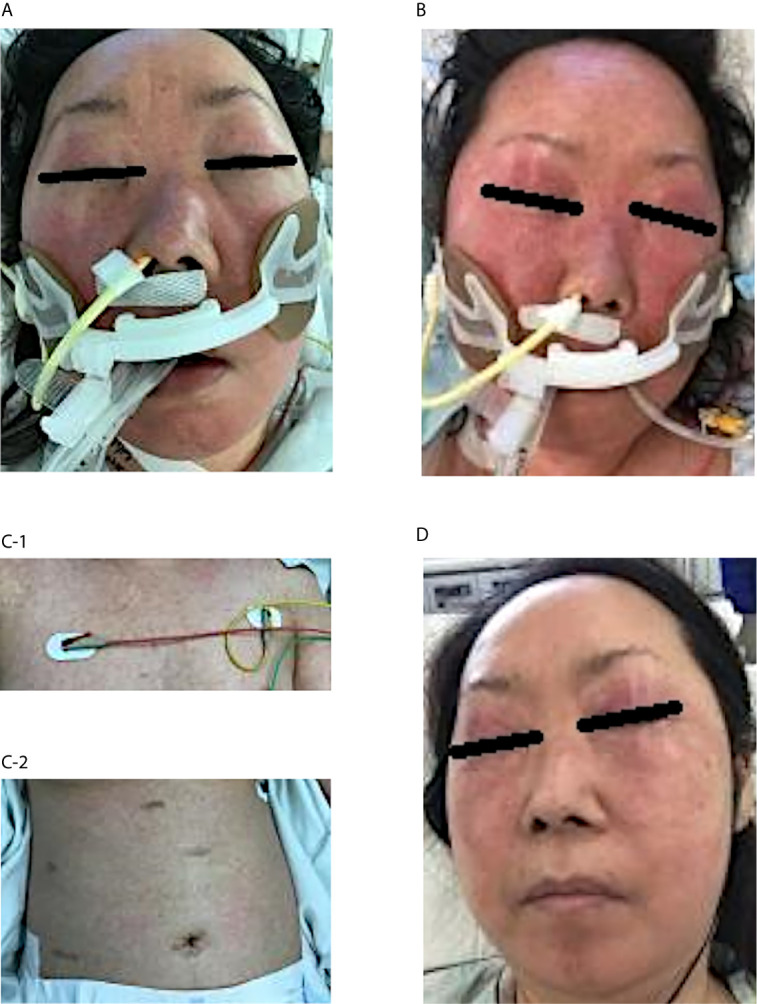
Clinical course of the skin rash after admission to the intensive care unit Edematous erythema appeared on the face, especially highlighted in the periorbital and perioral regions on Day 3 **(A)**. The skin rash was enhanced in spite of high-dose steroid therapy between Days 3 and 5 **(B)**. A maculopapular rash spread through the chest **(C-1)** and abdomen **(C-2)** on Day 7. The condition gradually disappeared by Day 12 after intravenous immunoglobulin therapy and subsequent steroid mini-pulse therapy **(D)**.

In the morning of the day that hypotensive shock occurred (Day 0), she had fever of 39.2°C with a transient skin rash, nausea, and vomiting. Subsequently, her blood pressure dropped to 72/46 mmHg. Although a large amount of fluid was rapidly infused intravenously, she remained hypotensive. Due to subsequent respiratory distress, she was intubated and underwent mechanical ventilation at the intensive care unit. Meanwhile, her blood pressure was maintained with continuous intravenous administration of norepinephrine (0.05-0.15 mg/kg/min). She also had an elevated number of white blood cells, increased C-reactive protein and hepatic enzyme serum levels, a decreased platelet count, and a coagulation abnormality (**Figure 1**). Although TMP/SMX was discontinued on Day 1, a maculopapular rash around the mouth strengthened and spread to the entire face, neck, trunk, and extremities in parallel with elevated levels of hepatobiliary enzymes on Days 3 and 5 (**Figure 1**). Edematous erythema, highlighted focally in the periorbital and perioral regions, was enhanced in spite of high dose steroids, between Days 3 (**Figure 2A**) and 5 (**Figure 2B**), and subsequently spread through the chest (**Figure 2C-1**) and abdomen (**Figure 2C-2**). All acute events that occurred simultaneously on Day 0 were considered to indicate life-threatening CRS (classified as Grade 4 according to the Common Terminology Criteria for Adverse Events, version 5.0) induced by ICIs, which would be steroid-resistant, rather than sepsis.

We administered tocilizumab (480 mg [8 mg/kg] in 250 mL normal saline intravenously for 2 h), a humanized anti-human interleukin (IL)-6 receptor (IL-6R) monoclonal antibody that blocks the signaling originated by the IL-6/IL-6R complex, to treat CRS and the steroid-resistant rash. The serum creatinine level increased day-by-day during the clinical course, indicating severe acute kidney injury. To remove waste and excess water, she received a total of four hemodialysis treatments. Because she was also suspected of having thrombotic thrombocytopenic purpura due to fever, decreased platelet count, and severe renal dysfunction, she received a plasma exchange. Although thrombotic thrombocytopenic purpura was excluded by ADAMTS13 (a disintegrin-like and metalloproteinase with thrombospondin type 1 motifs 13) testing, she was positive for heparin-induced thrombocytopenia antibodies. She received five days of intravenous immunoglobulin (IVIg) treatment (20 g/day [400 mg/kg/day], for 5 days). Subsequently, mini-pulse therapy using methylprednisolone (500 mg/day, for 3 days) was performed, followed by 50 mg/day (1 mg/kg/day) of prednisolone. She had a good response to these treatments and recovered well from her severe condition (**Figure 2D**).

Interestingly, the pulmonary metastatic lesions demonstrated pseudoprogression before the interstitial lung disease induced by ICIs, and thereafter they shrank and keep shrunk (**Supplementary Figure 1**).

## Discussion

To the best of our knowledge, this is the first report of a patient with TMP/SMX-triggered hypotensive shock related to the concomitant appearance of CRS, as an irAE, and DiHS, as an allergic reaction. TMP/SMX is well-known to induce allergic reactions such as fever and a subsequent rash 1 to 2 weeks after initial administration (2). In our case, high fever and a transient skin rash presented on the 11^th^ day of TMP/SMX administration. Interestingly, the eosinophil count increased despite high-dose steroid treatment immediately before the hypotensive shock (**Figure 1**). From the perspective that the peripheral eosinophil count generally decreases under high-dose corticosteroid treatment, this phenomenon appears to be a predictive sign of a severe irAE. Because both CRS and DiHS occurred at the time that TMP/SMX was likely to induce allergic reactions, the administration of TMP/SMX was considered to have begun too early.

In this case, it is probable that CRS, as an irAE, played a key role in hypotensive shock. CRS can present with a variety of symptoms ranging from mild to severe (3). Severe cases are characterized by high fever and hypotension, requiring vasopressors to maintain circulation. According to the laboratory data immediately following shock, the number of white blood cells and C-reactive protein level were extremely elevated (**Figure 1**). Shock liver (markedly elevated serum transaminases), renal dysfunction, and a coagulation abnormality also presented. Moreover, pro-inflammatory cytokines, such as serum levels of IL-6, tumor necrosis factor-α, interferon-γ, and the chemokine, monocyte chemoattractant protein-1, were elevated in our case (**Supplementary Table 1**). These factors are typically common in patients with CRS. However, serum levels of these cytokines were quite low compared to those previously reported (3–7). It is likely that the results did not reflect the actual situation. It is difficult to collect blood samples under the optimal conditions. We treated our patient with corticosteroids, an anti-IL-6 monoclonal antibody, hemodialysis, plasma exchange, and IVIg, and succeeded in recovering her condition. Because the pathophysiology of CRS is not fully understood (3), further studies regarding this syndrome are needed to implement more effective treatment strategies.

Characteristically of DiHS, the patient’s face exhibited edematous erythema, especially highlighted on the forehead and cheeks (**Figure 2**). Hypotensive shock occurred immediately after a fever of over 38°C, followed by liver abnormalities (alanine aminotransferase > 100 U/L) and leukocytosis (> 11 × 10^9^/L) (**Figure 1**). Our case did not fully meet the diagnostic criteria for DiHS established by a Japanese consensus group because of lacking evident lymphadenopathy and possible human herpesvirus-6 reactivation (4). Moreover, the maculopapular rash developed within 3 weeks after initiating TMP/SMX treatment, which is apart from the criteria. However, because our case had severe hepatic and renal dysfunction with a characteristic facial rash, we diagnosed the condition as atypical DiHS.

Previously reported ICI-related CRS cases are summarized in **Table 1** (5–8). In almost all cases, immunosuppressive therapies using corticosteroids were implemented. Moreover, in two cases of hypotensive shock requiring intubation and mechanical ventilation, additional agents, such as tocilizumab, mycophenolate mofetil, and IVIg, were administered for steroid-refractory symptoms (9, 10). Only our case was complicated with DiHS, probably induced by TMP/SMX.

**Table 1:** Immune checkpoint inhibitor-related cytokine release syndrome cases.

Authors	Age/Sex	Primary Cancer	ICI therapy	Clinical symptoms	Immunosuppressive therapy (highest dose)	Intervention except for immunosuppression	Outcome	Ref.
Rotz et al.	29/F	ASPS	Niv	[3 days before] Severe fatigue, myalgias, fever, diffuse maculopapular rash, subsequent encephalopathy [On presentation] hyperpyrexia, tachycardia, hypotension, hypoxia, disorientation, thrombocytopenia, acute kidney injury, liver enzyme elevation, CRP elevation	HDC (50 mg/m^2^/day), mPSL (2 mg/kg/day), Tocilizumab (1st.: 4 mg/kg, 2nd.: 8 mg/kg)	Not particular	Recovered	(5)
Dimitriou et al.	47/M	Melanoma	Pem/Epa	Pyrexia, chills, diffuse maculopapular skin rash, hypotension, tachycardia, ventricular extrasystoles, renal insufficiency, liver abnormalities	mPSL (250 mg/day), PSL (50 mg/day)	Not particular	Recovered	(6)
Dimitriou et al.	48/F	Melanoma	Pem/T-VEC, Niv/BMS	[1st.] Generalized maculopapular rash, pyrexia, chills, tachycardia, hypotension, liver abnormalities [2nd.] pyrexia, tachycardia, leukopenia, transient generalized macular rash, oral erosive mucositis [3rd.] pyrexia, tachycardia, skin rash with blisters and bullae, erosive stomatitis	PSL (80 mg [1 mg/kg/day]/day), mPSL (250 mg/day), Tocilizumab	Not particular	Recovered	(6)
Oda et al.	43/M	Gastric cancer	Niv	Fever, tachycardia, appetite loss, malaise, elevated levels of bilirubin, liver enzyme, biliary enzyme and CRP	PSL (1 mg/kg/day), mPSL pulse therapy (1 g/day, for 3 days), MMF (2000 mg/day)	Not particular	Died of cancer	(8)
Honjo et al.	52/F	PCL	Niv	Asthenia, fever, livedo reticularis on the extremities with systemic purpura, thrombocytopenia, increased triglycerides, impaired liver function, increased creatine phosphokinase, muscle soreness, cardiac hypofunction, respiratory distress, renal failure, gangrene in the lower extremities	mPSL pulse therapy (1 g/day, for 3 days), PSL (50 mg/day), MMF	CHDF, amputation of both lower extremities	Recovered	(7)
Adashek et al.	72/M	LAD		Hypotension, respiratory distress	Tocilizumab	Intubation	Recovered	(9)
Ohira et al.	70/M	RCC	Niv/Ipi	Erythema, muscle weakness, fever, hypotension, respiratory failure, impaired consciousness, renal failure, coagulation abnormality, oral enanthema, diarrhea	PSL (60 mg/day), mPSL pulse therapy (500 mg/day, for 3 days), MMF (2000 mg/day), IVIg	Intubation, CHDF, PE, HD	Recovered	(10)
Our case	46/F	RCC	Niv/Ipi 4 cycles	Hypotension, respiratory failure, shock liver, platelet decreased, renal failure, coagulation abnormality, rash	PSL (100 mg [2 mg/kg]/day), HDC (300 mg/day), mPSL pulse therapy (500 mg/day, for 3 days), Tocilizumab, IVIg (for 5 days)	Intubation, HD, PE	Recovered	

ASPS, alveolar soft part sarcoma; HL, Hodgkin’s lymphoma; PCL, pleomorphic cancer of the lung; LAD, lung adenocarcinoma; RCC, renal cell carcinoma; Niv, nivolumab; Pem, pembrolizumab; Epa, epacadostat; BMS, BMS-986016 (anti-LAG-3 antibody); CRP, C-reactive protein; PSL, prednisolone; mPSL, methylprednisolone; MMF, mycophenolate mofetil; IVIg, intravenous immunoglobulin; HDC, hydrocortisone sodium succinate; CHDF, continuous hemodiafiltration; HD, hemodialysis; PE, plasma exchange.

## Conclusion

The prophylactic administration of TMP/SMX should be performed cautiously to avoid severe irAEs and allergic reactions, such as CRS and DiHS, respectively.

## Data Availability Statement

The original contributions presented in the study are included in the article/**Supplementary Material**. Further inquiries can be directed to the corresponding author.

## Ethics Statement

Written informed consent was obtained from the individual(s) for the publication of any potentially identifiable images or data included in this article.

## Author Contributions

TU drafted the manuscript. TU, MO, TM, KT, and AN contributed to the management of the clinical case and interpretation of clinical data. EF, MF, YK, and TY contributed to the patient’s care. MO, TM, KT, AN, SK, and TY reviewed the manuscript. SK, JY, and ST supervised this study. All authors contributed to read and approved the final manuscript.

## Conflict of Interest

The authors declare that the research was conducted in the absence of any commercial or financial relationships that could be construed as a potential conflict of interest.

## Supplementary Material

Supplementary Figure 1 Axial computed tomographic scan images of the chest showing metastatic pulmonary lesions. Upper column **(A–E)**: lesion #1 (right pulmonary S5, yellow arrowhead), lower column **(F–J)**: lesion#2 (right pulmonary S8, white arrowhead). **(A, F)** Baseline (1 day before laparoscopic total right nephrectomy). **(B, G)** After 2 cycles of nivolumab plus ipilimumab combination therapy. **(C, H)** On the day of the appearance of interstitial lung diseases induced by immune checkpoint inhibitors. **(D, I)** On the day of hypotensive shock. **(E, J)** After recovery from cytokine release syndrome and drug-induced hypersensitivity syndrome (steroid-free). Lesion #1 was not changed in size after 2 cycles of nivolumab plus ipilimumab combination therapy **(A, B)**, however the lesion was enlarged on the day of appearance of interstitial lung disease induced by the immune checkpoint inhibitor (ICI) combination therapy **(C)**. This phenomenon was considered so-called pseudoprogression based on the accumulation of lymphocytes to the metastatic lesion, induced by the ICI therapy. Thereafter, the lesion shrank **(D)**, and its size was maintained without any treatments **(E)**. Lesion #2 had pseudoprogression after 2 cycles of nivolumab plus ipilimumab combination therapy **(F, G)**. This lesion shrank on the day of appearance of interstitial lung disease with the ICI combination therapy **(H)** and continued to shrink without any treatments **(I, J)**.
