# Supplementary figures and images for: Case Report: A Case of Trimethoprim/Sulfamethoxazole-Triggered Hypotensive Shock: Cytokine Release Syndrome Related to Immune Checkpoint Inhibitors and Drug-Induced Hypersensitivity Syndrome
Source: Front Oncol. 2021 Apr 30;11:681997. doi: 10.3389/fonc.2021.681997 (PMC8121494; doi:10.3389/fonc.2021.681997)

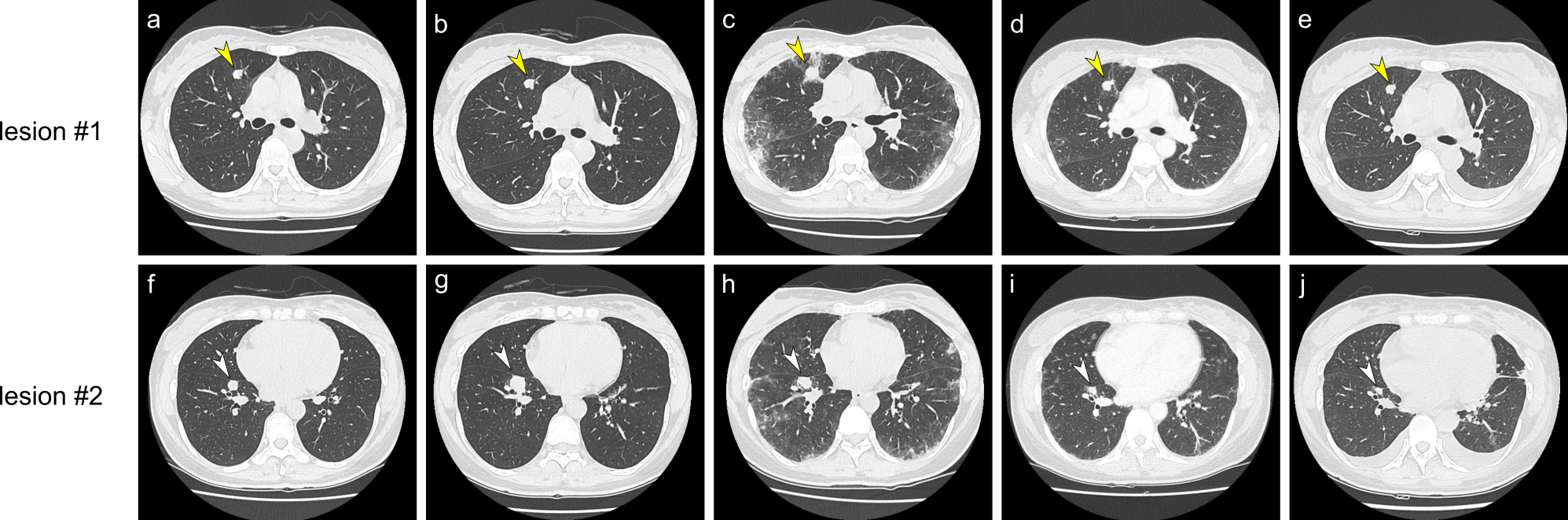

Supplement: Supplementary Figure 1 — Axial computed tomographic scan images of the chest showing metastatic pulmonary lesions. Upper column (A–E): lesion #1 (right pulmonary S5, yellow arrowhead), lower column (F–J): lesion#2 (right pulmonary S8, white arrowhead). (A, F) Baseline (1 day before laparoscopic total right nephrectomy). (B, G) After 2 cycles of nivolumab plus ipilimumab combination therapy. (C, H) On the day of the appearance of interstitial lung diseases induced by immune checkpoint inhibitors. (D, I) On the day of hypotensive shock. (E, J) After recovery from cytokine release syndrome and drug-induced hypersensitivity syndrome (steroid-free). Lesion #1 was not changed in size after 2 cycles of nivolumab plus ipilimumab combination therapy (A, B), however the lesion was enlarged on the day of appearance of interstitial lung disease induced by the immune checkpoint inhibitor (ICI) combination therapy (C). This phenomenon was considered so-called pseudoprogression based on the accumulation of lymphocytes to the metastatic lesion, induced by the ICI therapy. Thereafter, the lesion shrank (D), and its size was maintained without any treatments (E). Lesion #2 had pseudoprogression after 2 cycles of nivolumab plus ipilimumab combination therapy (F, G). This lesion shrank on the day of appearance of interstitial lung disease with the ICI combination therapy (H) and continued to shrink without any treatments (I, J). [file Image_1.tif]
