# Supplementary material for: Case Report: A Case of Trimethoprim/Sulfamethoxazole-Triggered Hypotensive Shock: Cytokine Release Syndrome Related to Immune Checkpoint Inhibitors and Drug-Induced Hypersensitivity Syndrome
Source: Front Oncol. 2021 Apr 30;11:681997. doi: 10.3389/fonc.2021.681997 (PMC8121494; doi:10.3389/fonc.2021.681997)
Supplement: Supplementary file 2 [file Presentation_1.pdf]

Figure Legend

Supplementary Figure 1 | Axial computed tomographic scan images of the chest showing metastatic pulmonary lesions. Upper column **(a-e)**: lesion #1 (right pulmonary S5, yellow arrowhead), lower column **(f-j)**: lesion#2 (right pulmonary S8, white arrowhead). **a, f.** Baseline (1 day before laparoscopic total right nephrectomy). **b, g.** After 2 cycles of nivolumab plus ipilimumab combination therapy. **c, h.** On the day of the appearance of interstitial lung diseases induced by immune checkpoint inhibitors. **d, i.** On the day of hypotensive shock. **e, j.** After recovery from cytokine release syndrome and drug-induced hypersensitivity syndrome (steroid-free).

Lesion #1 was not changed in size after 2 cycles of nivolumab plus ipilimumab combination therapy (**a, b**), however the lesion was enlarged on the day of appearance of interstitial lung disease induced by the immune checkpoint inhibitor (ICI) combination therapy (**c**). This phenomenon was considered so-called pseudoprogression based on the accumulation of lymphocytes to the metastatic lesion, induced by the ICI therapy. Thereafter, the lesion shrank (**d**), and its size was maintained without any treatments (**e**).

Lesion #2 had pseudoprogression after 2 cycles of nivolumab plus ipilimumab combination therapy (**f, g**). This lesion shrank on the day of appearance of interstitial lung disease with the ICI combination therapy (**h**) and continued to shrink without any treatments (**i, j**).
