# Supplementary material for: Case Report: A Case of Trimethoprim/Sulfamethoxazole-Triggered Hypotensive Shock: Cytokine Release Syndrome Related to Immune Checkpoint Inhibitors and Drug-Induced Hypersensitivity Syndrome
Source: Front Oncol. 2021 Apr 30;11:681997. doi: 10.3389/fonc.2021.681997 (PMC8121494; doi:10.3389/fonc.2021.681997)
Supplement: Supplementary file 3 [file Table_1.pdf]

Supplementary Table 1. Cytokine values in cytokine release syndrome cases

	Rotz *et al.* [3]	Dimitriou *et al.* [4] Case 1	Dimitriou *et al.* [4] Case 2	Oda *et al.* [5]	Honjo *et al.* [6]	Adashek *et al.* [7]	Ohira *et al.* [8]	Our case
IL-1β (pg/ml)	16	N/A	N/A	N/A	**34.9**	N/A	**7.41**	< 0.125 (≤0.928)
IL-2 (pg/ml)	3	N/A	N/A	Not elevated	< 15.6	N/A	< 15.6	N/A
IL-6 (pg/ml)	**45**	**66.4**	**[1st.] 38.4 [2nd.] > 300 [3rd.] 67.5**	**> 100**	**1,510**	N/A	**467**	**5.6 (before tocilizumab) 55.7 (after tocilizumab)** (0.0~4.0)
IL-8 (pg/ml)	**125**	N/A	N/A	N/A	N/A	N/A	N/A	N/A
IL-10 (pg/ml)	**128**	N/A	N/A	N/A	**20.0**	N/A	**40.6**	≤2 (≤5)
IL-18 (pg/ml)	N/A	N/A	N/A	N/A	N/A	N/A	**2,510**	N/A
IFN-γ (pg/ml)	**660**	**312.7**	**[1st.] 143.2 [2nd.] > 1,000 [3rd.] 2,236**	**> 300**	**9,260**	N/A	**1,020**	**6.4 IU/mL** (≤0.1)
TNF-α (pg/ml)	3	6.50	**[2nd.] 15.6** [3rd.] 5.73	**> 300**	**251**	N/A	**47.7**	**5.44** (0.75~1.66)
MCP-1 (pg/ml)	N/A	N/A	N/A	N/A	N/A	N/A	N/A	**391** (≤149)
GM-CSF (pg/ml)	1	N/A	N/A	N/A	N/A	N/A	N/A	N/A
G-CSF (pg/ml)	N/A	N/A	N/A	N/A	**753**	N/A	N/A	N/A
ADAMTS13 activity (%)	N/A	N/A	N/A	N/A	37.8	N/A	N/A	90
ADAMTS13 inhibitor (BU/ml)	N/A	N/A	N/A	N/A	< 0.5	N/A	N/A	< 0.5
Ferritin (ng/ml)	N/A	N/A	N/A	**> 600**	**3,877**	N/A	N/A	**3169.2** (10.0~200.0)
sIL-2R (U/ml)	N/A	N/A	N/A	N/A	**3,630**	**N/A**	**5,764**	**9,054** (145~519)

Bold letters indicate values above the normal range.

IL, interleukin; IFN-γ, interferon-γ; TNF-α, tumor necrosis factor-α; MCP-1, monocyte chemoattractant protein-1; GM-CSF, granulocyte-macrophage colony-stimulating factor; ADAMTS13, a disintegrin-like and metalloproteinase with thrombospondin type 1 motifs 13, BU, Bethesda unit; sIL-2R, soluble interleukin-2 receptor, N/A, not available
